# The prevalence of low muscle mass associated with obesity in the USA

**DOI:** 10.1186/s13395-022-00309-5

**Published:** 2022-12-21

**Authors:** Dana J. Murdock, Ning Wu, Joseph S. Grimsby, Roberto A. Calle, Stephen Donahue, David J. Glass, Mark W. Sleeman, Robert J. Sanchez

**Affiliations:** Regeneron Pharmaceuticals, Inc., 777 Old Saw Mill River Road, Tarrytown, NY 10591-6707 USA

**Keywords:** Sarcopenic obesity, Sarcopenia, Prevalence, Body mass index, Atrophy, Muscle loss

## Abstract

**Background:**

Sarcopenia is defined as age-related low muscle mass and function, and can also describe the loss of muscle mass in certain medical conditions, such as sarcopenic obesity. Sarcopenic obesity describes loss of muscle and function in obese individuals; however, as sarcopenia is an age-related condition and obesity can occur in any age group, a more accurate term is obesity with low lean muscle mass (OLLMM). Given limited data on OLLMM (particularly in those aged < 65 years), the purpose of this study was to estimate the prevalence of OLLMM in adults aged ≥ 20 years in the USA.

**Methods:**

Data from the National Health and Nutrition Examination Survey (NHANES) 2017–2018 and 1999–2006 were used. OLLMM was defined as an appendicular lean mass, adjusted for body mass index (BMI), cut-off point < 0.789 for males and < 0.512 for females, measured by dual-energy X-ray absorptiometry (DXA). DXA was only measured in individuals 20–59 years old in NHANES 2017–2018; we therefore utilized logistic regression models to predict OLLMM from NHANES 1999–2006 for those aged ≥ 60 years. The prevalence of OLLMM was estimated overall, and by sex, age, race/ethnicity, and clinical subgroup (high BMI, prediabetes, type 2 diabetes mellitus [T2DM], non-alcoholic fatty liver disease [NAFLD] with fibrosis, or post-bariatric surgery). Prevalence estimates were extrapolated to the USA population using NHANES sampling weights.

**Results:**

We estimated that, during 2017–2018, 28.7 million or 15.9% of the USA population had OLLMM. The prevalence of OLLMM was greater in older individuals (8.1%, aged 20–59 years vs 28.3%, aged ≥ 60 years), highest (66.6%) in Mexican-American females aged ≥ 60 years, and lowest (2.6%) in non-Hispanic Black males aged 20–59 years. There was a higher prevalence of OLLMM in adults with prediabetes (19.7%), T2DM (34.5%), NAFLD with fibrosis (25.4%), or post-bariatric surgery (21.8%), compared with those without each condition.

**Conclusions:**

Overall, the burden of OLLMM in the USA is substantial, affecting almost 30 million adults. The prevalence of OLLMM increased with age, and among those with prediabetes, T2DM, NAFLD with fibrosis, or post-bariatric surgery. A unified definition of OLLMM will aid diagnosis and treatment strategies.

**Supplementary Information:**

The online version contains supplementary material available at 10.1186/s13395-022-00309-5.

## Background

The prevalence of obesity is increasing globally, with numbers doubling in more than 70 countries since 1980 [1]. Among children and adolescents, the prevalence of obesity has more than tripled since the 1970s [2, 3]. These dramatic increases in prevalence have led to a rise in obesity-associated comorbidities [4].

Sarcopenia, defined as loss of skeletal muscle mass and function in older people [5] has recently been classified as a muscle disease, and is diagnosed based on an assessment of muscle mass, strength, and anthropometric measures [6–9]. The term sarcopenia has also been used to denote loss of muscle due to a particular clinical comorbidity; for example “sarcopenic obesity,” which describes muscle loss in obese individuals [10, 11]. However, since the term sarcopenia is considered age-related and obesity can occur at any age, we proposed using the term obesity with low lean muscle mass (OLLMM) to describe this phenomenon across all age groups. OLLMM has been proposed to occur as a result of mechanistic inflammatory signaling, activin, and metabolic syndrome pathway activation by adipose-produced cytokines, ultimately increasing catabolism of lean muscle [12–14]. It is characterized by the co-existence of reduced muscle mass and excess fat mass. Although OLLMM can occur at any age, it is more common in older adults [15]. Both obesity and sarcopenia are independently associated with chronic cardiovascular diseases and diabetes [16, 17]. Loss of muscle in obese individuals has been linked to increased insulin resistance, risk of type 2 diabetes mellitus (T2DM), disability, morbidity, and mortality [16, 18–21]. Sarcopenia has been associated with insulin resistance, liver fibrosis [22, 23], frailty, and mortality [24]. Sarcopenia and obesity both display distinct and common pathophysiological features, which may act synergistically [16, 18] to increase the risk of developing adverse health issues. Consequently, people with both sarcopenia and obesity are an especially vulnerable population. Therefore, it is important to identify affected individuals and understand the factors that could contribute to morbidity within this population [19].

Historically, the prevalence of sarcopenic obesity has been difficult to assess, due to the unclear and confusing nature of the term, as well as a paucity of data for younger age groups. As such, prevalence estimates vary dramatically between different studies due to heterogeneity in the definitions of sarcopenia and obesity, as well as the populations assessed [25, 26]. Previous prevalence estimates of sarcopenic obesity in the USA, obtained from National Health and Nutrition Examination Survey (NHANES) data, are limited. Several of the prior estimates rely on data roughly 20 years old [27–29]. Additionally, the estimate using more recent data (NHANES 2017–2018) does not include participants aged > 59 years, thus excluding the population at the highest risk for sarcopenic obesity [30].

The lack of a standard definition makes the condition difficult to diagnose (impacting prevalence estimates and development of treatment strategies) and can result in mixed populations in clinical trials [6, 15, 31]. A consensus on diagnostic tools and criteria is needed, as well as identification of optimal prevention and treatment options [32]. The objective of this study was to estimate the prevalence of low muscle mass in obese individuals (previously known as sarcopenic obesity), in USA adults aged ≥ 20 years. Additionally, we assessed how the prevalence of OLLMM differed by age, race/ethnicity, high-risk clinical subgroups, and body mass index (BMI) categories.

## Methods

### Data sources and analytic sample

A cross-sectional multi-step analysis was performed using data from NHANES 1999–2006 and 2017–2018. The analytic sample included all survey participants who were aged ≥ 20 years. NHANES uses a complex, multistage probability sampling design to select participants representative of the non-institutionalized USA population, and collects demographic, socioeconomic, dietary, and health-related data. Further details of the NHANES methods and analytic guidelines are described elsewhere [33].

### Measurement and definition of OLLMM

Dual-energy X-ray absorptiometry (DXA) measurements (bone and soft tissue) of body composition for survey participants have been collected as part of the NHANES study since 1999. DXA is considered a robust and reproducible way of measuring components of body composition, including bone density, body fat (BF) percentage, and appendicular lean mass (ALM) [6]. In the 2017–2018 survey, DXA data were available for participants aged 20–59 years. BMI was calculated from height and weight measurements, which were collected by trained health technicians and were available for all ages. In order to determine OLLMM in participants ≥ 60 years of age, data from prior years of NHANES were used, when DXA was measured across a wider range of ages (i.e., ages ≥ 8 years in 1999–2004, and ages 8–69 years in 2005–2006), to construct models predicting OLLMM among those aged ≥ 60 years in the 2017–2018 dataset described in detail below.

OLLMM was defined using the Foundation for the National Institutes of Health (FNIH) criteria for low lean mass associated with weakness (ALM adjusted for BMI, cut-off point for males < 0.789 and for females < 0.512) [34, 35], and the American Association of Clinical Endocrinology BF percentage thresholds for the diagnosis of obesity (males > 25% and females > 35%) [36].

### Predicting OLLMM and sarcopenia in patients aged ≥ 60 years

For individuals aged ≥ 60 years, OLLMM status was predicted using an algorithm, modeled using various measures based on the 1999–2006 NHANES data, and then applied to the 2017–2018 NHANES data. The sensitivity analysis defined OLLMM based on ALM and BMI ≥ 27 kg/m^2^ [36]. A BMI of ≥ 27 kg/m^2^ was chosen to define OLLMM, as it is the minimum BMI threshold recommended for intervention with anti-obesity medications by the Obesity Society, the Endocrine Society, and the American Association of Clinical Endocrinologists [36–38].

### Definition of other measures

Participants were stratified (Supplemental Table﻿ S1) by high BMI (class 1: BMI 30 to < 35 kg/m^2^, class 2: BMI 35 to < 40 kg/m^2^, class 3: BMI ≥ 40 kg/m^2^); prediabetes (based on glycated hemoglobin ≥ 5.7% to < 6.5%, or if the participant responded yes to the question “Have you ever been told by a doctor or other health professional that you have any of the following: prediabetes, impaired fasting glucose, impaired glucose tolerance, or borderline diabetes, or that your blood sugar is higher than normal but not high enough to be called diabetes or sugar diabetes?”); T2DM (based on glycated hemoglobin ≥ 6.5%, or answered yes to “Other than during pregnancy, have you ever been told by a doctor or health professional that you have diabetes or sugar diabetes?”), excluding those who reported insulin therapy only or were diagnosed with diabetes aged < 30 years [39]; non-alcoholic fatty liver disease (NAFLD) with fibrosis (based on median controlled attenuated parameter of > 285 dB/m, and liver stiffness of ≥8 kPa [excluding those with excessive alcohol consumption determined using self-reported average number of alcohol drinks/day over the past 12 months]); and history of bariatric surgery (based on those who answered yes to “Have you ever had weight loss surgery?”).

### Statistical analysis

Descriptive analyses of baseline characteristics and outcome variables were performed. Median values (25th and 75th percentile) are presented for continuous outcomes. Frequency, weighted frequency, and weighted proportion (95% confidence intervals [CIs]) are presented for categorical measures.

The NHANES 1999–2006 data were split into a 70% training sample and a 30% testing sample. Logistic regression was used to create age (< 60 and ≥ 60 years) and sex-specific models utilizing the training sample, as the mechanism and predictors were expected to be different. Backward stepwise selection was used to identify potential predictors, such as demographics (age, sex, race/ethnicity, region), comorbidities, anthropometric measures, laboratory results, and physical performance measures were identified from prior literature. Variables that were consistently significant (*p* < 0.1) across different models were selected for the final model. Performance of the fitted models was tested on the 30% validation sample. The area under the receiver operating characteristic curve (AUC) was calculated to evaluate the model performance in the training and testing samples. Once a high-performing model was selected, it was used to predict the likelihood that an individual (aged ≥ 60 years) in the 2017–2018 survey year had OLLMM. For further details on model performance, please see the Supplementary material and Supplemental Table﻿ S2.

Data were analyzed using SAS version 9.4 (SAS Institute, Cary, NC, USA). Survey procedures were used to account for the complex NHANES survey design, the sampling weights and non-response. Prevalence was estimated based on the number of cases of OLLMM identified via DXA (patients aged 20–59 years) or the classification model (patients aged > 60 years). Analytical methods accounting for multiple imputing and combined sampling weight across the four waves of data were used when analyzing NHANES 1999–2006 data [40].

## Results

A total of 9254 participants were included in the 2017–2018 NHANES survey; 4174 of those were ≥ 20 years and eligible for this study. Of the individuals included, 2156 (weighted percentage 61.6%) were aged 20–59 years with DXA scans, and 2018 (weighted percentage 38.4%) were aged ≥ 60 years with predicted OLLMM status (Table 1). Approximately half the participants were female (2118, 52.0%). Sample sizes within each age and race/ethnic group are listed in Table 1. The prediction model was developed using data from 4889 participants aged ≥ 60 years from the 1999–2006 NHANES database (see Supplementary material), and model performance for OLLMM was excellent for both males and females aged ≥ 60 years, with an AUC of 0.91 and 0.87, respectively (Supplemental Figures S1 and S2, and Supplemental Table S2).

**Table 1:** Prevalence of OLLMM in different groups (based on percentage BF)

Variable	With OLLMM, ***n***	Eligible participants, ***n***	With OLLMM, weighted, ***n***	Eligible participants, weighted, ***n***	Prevalence OLLMM,^**a**^ % (95% CI)
All	827	4174	28,728,420	181,176,597	15.9 (13.7–18.0)
Age group, years					
20–29	36	536	1,591,786	31,069,719	5.1 (2.1–8.1)
30–39	35	522	1,735,137	27,141,372	6.4 (3.6–9.2)
40–49	64	517	2,198,743	25,608,035	8.6 (5.8–11.3)
50–59	95	581	3,475,076	27,761,724	12.5 (8.1–17.0)
60–69	263	1057	7,465,838	36,875,173	20.2 (14.8–25.7)
70–79	202	579	7,980,944	21,671,913	36.8 (31.7–42.0)
≥ 80	132	382	4,280,896	11,048,661	38.7 (32.3–45.2)
Sex					
Males	409	2056	13,273,570	86,966,677	15.3 (12.3–18.2)
Females	418	2118	15,454,850	94,209,920	16.4 (14.0–18.8)
Race/ethnicity^b^					
Mexican-American	196	549	4,153,037	15,390,072	27.0 (22.7–31.3)
Other Hispanic	112	401	2,688,457	12,765,397	21.1 (16.4–25.7)
Non-Hispanic White	355	1496	18,565,102	115,853,319	16.0 (13.1–18.9)
Non-Hispanic Black	42	899	670,921	17,954,423	3.7 (2.5–5.0)
Other, including multi-racial	122	829	2,650,903	19,213,386	13.8 (10.2–17.4)
OLLMM based on BMI^c^					
No	196	3503	5,666,439	157,242,384	3.6 (2.9–4.3)
Yes	631	671	23,061,982	23,934,214	96.4 (95.0–97.7)
Age vs sex					
Females aged 20–59 years	108	1122	3,774,244	56,255,046	6.7 (4.4–9.0)
Females aged ≥ 60 years	310	996	11,680,606	37,954,874	30.8 (26.3–35.2)
Males aged 20–59 years	122	1034	5,226,498	55,325,803	9.4 (6.9–12.0)
Males aged ≥ 60 years	287	1022	8,047,072	31,640,874	25.4 (19.6–31.2)

*ALM* appendicular lean mass, *BF* body fat, *BMI* body mass index, *CI* confidence interval, *NHANES* National Health and Nutrition Examination Survey, *OLLMM* obesity with low lean muscle mass

^a^OLLMM based on ALM and percentage BF

^b^Race/ethnicity based on NHANES categories

^c^Individuals identified with OLLMM, using BMI ≥ 27 kg/m^2^ to define obesity, were also identified based on the percentage BF criteria

### Overall prevalence

Of the 4174 participants who were included in the study (representing 181,176,597 individuals aged ≥ 20 years in the USA), 827 (representing 28,728,420 individuals) were classified as having OLLMM, either using the prediction model for those aged ≥ 60 years or directly from the 2017–2018 NHANES data for those aged 20–59 years. The overall prevalence of OLLMM was estimated at 15.9% (95% CI, 13.7–18.0%) of the USA population (Table 1).

### Prevalence of OLLMM by sex, age group, and race

The prevalence of OLLMM was 16.4% (95% CI, 14.0–18.8%) and 15.3% (95% CI, 12.3–18.2%) in females and males, respectively (Table 1). Prevalence of OLLMM increased with each decade of life starting as low as 5.1% in those aged 20–29 years and increasing to 38.7% among those aged ≥80 years (Table 1). When stratified by 20–59 and ≥ 60 years of age, the prevalence of OLLMM was greater in those aged ≥ 60 years (8.1% of those aged 20–59 years vs 28.3% of those aged ≥ 60 years). Additionally, compared with non-Hispanic Whites, OLLMM was higher in Mexican-Americans and other Hispanic ethnic groups (27.0%; 95% CI, 22.7–31.3%, and 21.1%; 95% CI, 16.4–25.7%, respectively), and lowest in non-Hispanic Black groups (3.7%; 95% CI, 2.5–5.0%; Table 1). Furthermore, in analyses stratified by age, sex, and race/ethnicity, the highest prevalence of OLLMM (66.6%; 95% CI, 59.8–73.4%) was found among Mexican-American females aged ≥ 60 years and the lowest prevalence (2.6%; 95% CI, 1.1–4.0%) was found in non-Hispanic Black males aged 20–59 years (Table 2).

**Table 2:** Prevalence of OLLMM^a^ by age, sex, and race/ethnicity (based on percentage BF)

% (95% CI)	Race/ethnicity				
	Mexican-American	Other Hispanic	Non-Hispanic White	Non-Hispanic Black	Other, including multi-racial
Males aged 20–59 years	18.3 (12.1–24.5)	18.9 (8.5–29.2)	7.0 (3.0–11.0)	2.6 (1.1–4.0)	11.3 (5.9–16.7)
Males aged ≥ 60 years	48.3 (37.2–59.5)	36.8 (25.1–48.4)	25.2 (18.0–32.5)	5.3 (2.7–7.9)	30.1 (17.1–43.1)
Females aged 20–59 years	20.9 (15.7–26.1)	10.4 (3.5–17.4)	4.3 (2.4–6.3)	3.0 (0.7–5.3)	6.4 (2.1–10.7)
Females aged ≥ 60 years	66.6 (59.8–73.4)	42.1 (33.9–50.3)	32.3 (26.8–37.8)	5.6 (2.5–8.7)	21.6 (12.2–31.0)

*ALM* appendicular lean mass, *BF* body fat, *CI* confidence interval, *OLLMM* obesity with low lean muscle mass

^a^OLLMM based on ALM and percentage BF

### Prevalence of OLLMM by clinical subgroups and BMI

The prevalence of OLLMM was higher in people with the following clinical conditions/procedures compared with those without: T2DM (34.5% vs 12.7%), prediabetic (19.7% vs 14.7%), those diagnosed with NALFD with fibrosis (25.4% vs 12.5%), or in those who had undergone bariatric surgery (21.8% vs 15.8%; Table 3). Additionally, we found that the prevalence of OLLMM increased as BMI increased, with 9.2% (95% CI, 7.8–10.6%) of those with BMI < 30 kg/m^2^ having OLLMM compared with 35.0% (95% CI, 26.1–43.9%) of those with BMI ≥ 40 kg/m^2^.

**Table 3:** Prevalence of OLLMM in different clinical subgroups (based on percentage BF)

Clinical subgroups	With OLLMM, ***n***	Eligible participants, ***n***	With OLLMM, weighted, ***n***	Eligible participants, weighted, ***n***	Prevalence OLLMM,^**a**^ % (95% CI)
T2DM, no	527	3277	19,589,822	154,709,933	12.7 (10.5–14.8)
T2DM, yes	300	897	9,138,598	26,466,664	34.5 (29.5–39.6)
Prediabetes, no	577	3060	20,645,798	140,079,916	14.7 (12.1–17.4)
Prediabetes, yes	250	1114	8,082,622	41,096,682	19.7 (16.6–22.7)
NAFLD with fibrosis, no	366	2408	14,954,200	119,957,621	12.5 (10.3–14.6)
NAFLD with fibrosis, yes	39	109	1,109,842	4,361,071	25.4 (15.7–35.2)
Bariatric surgery, no	811	4122	28,170,588	178,616,433	15.8 (13.5–18.0)
Bariatric surgery, yes	16	52	557,833	2,560,164	21.8 ﻿(8.6–35.0)
BMI, kg/m^2^					
< 30	340	2510	9,961,709	108,595,331	9.2 (7.8–10.6)
30 to < 35	240	896	8,553,115	40,325,428	21.2 (16.9–25.5)
35 to < 40	133	430	6,227,994	19,277,764	32.3 (25.9–38.7)
≥ 40	113	283	3,953,401	11,292,403	35.0 (26.1–43.9)

*ALM* appendicular lean mass, *BF* body fat, *BMI* body mass index, *CI* confidence interval, *NAFLD* non-alcoholic fatty liver disease, *OLLMM* obesity with low lean muscle mass, *T2DM* type 2 diabetes mellitus

^a^OLLMM based on ALM and percentage BF

### Characteristics of individuals with OLLMM

Among those with OLLMM, the majority were female (53.8%; 95% CI, 48.3–59.3%) and most (64.6%; 95% CI, 56.5–72.7%) were non-Hispanic White (Supplemental Table S3). The median age was higher among those with OLLMM at 65.2 years (interquartile range [IQR] 54.9–74.8) compared with 47.9 years (IQR 32.6–62.5) in those without OLLMM. Median BMI and waist circumference were higher in those with OLLMM compared with: those without 32.6 kg/m^2^ (IQR 28.6–36.3) and 108.9 cm (IQR 100.0–118.9) versus 27.4 kg/m^2^ (IQR 23.7–31.7), and 96.2 cm (IQR 85.7–107.4), respectively (Supplemental Table S3).

**Fig. 1:**
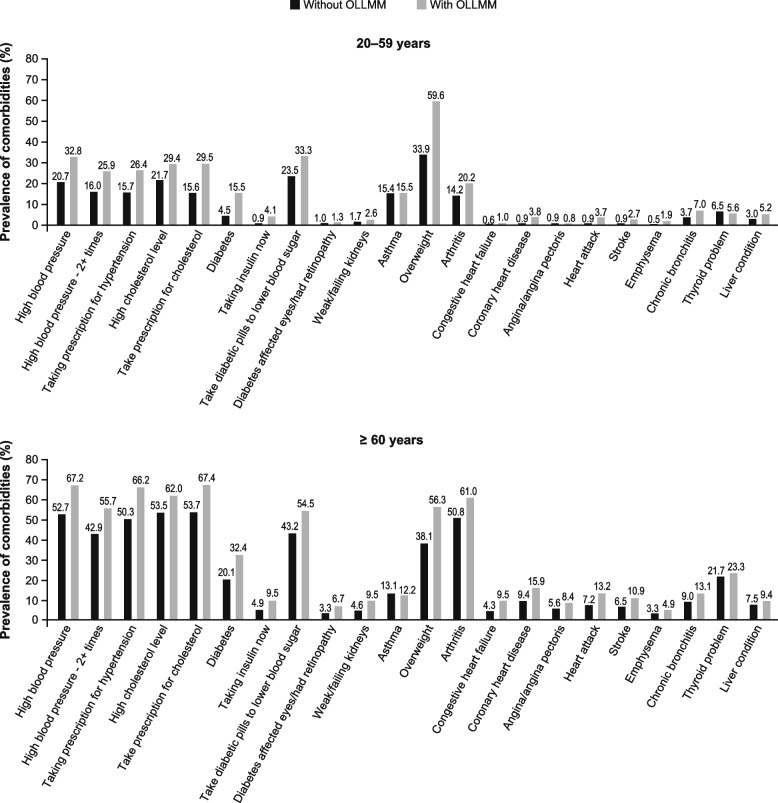
Comorbidities in participants with and without OLLMM (based on percentage BF). BF, body fat; OLLMM, obesity with low lean muscle mass

Comorbidities and general health were also associated with the prevalence of OLLMM. The prevalence of comorbidities, including high blood pressure or high cholesterol level, tended to be higher among those with OLLMM compared with those without (Fig. 1). A higher percentage of people with OLLMM reported poor or fair general health compared with those without OLLMM (Table 4). Across all age groups, those with OLLMM tended to have more physical limitations, with a higher percentage reporting limitations in stooping, crouching, kneeling, or standing for long periods (Supplemental Table S4).

**Table 4:** General health and clinical recommendations for participants with or without OLLMM (based on percentage BF)

	Without OLLMM	With OLLMM
Participants, weighted (*n*, unweighted)	152,448,177 (3347)	28,728,420 (827)
**Variable, % (95% CI)**		
What is your health in general?		
Excellent	10.8 (8.9–12.8)	6.8 (4.3–9.4)
Very good	34.8 (31.8–37.8)	19.3 (15.2–23.3)
Good	38.2 (35.8–40.6)	45.3 (40.1–50.6)
Fair	14.4 (12.3–16.5)	24.2 (19.9–28.5)
Poor	1.7 (1.2–2.3)	4.4 (2.7–6.1)
Has a doctor told you to?		
Lose weight	23.6 (21.0–26.2)	41.7 (34.2–49.1)
Increase exercise	35.5 (32.5–38.6)	56.9 (48.8–65.0)
Reduce salt intake	22.7 (19.6–25.8)	38.4 (31.9–44.9)
Reduce fat/calorie intake	27.0 (23.5–30.5)	43.0 (35.9–50.1)
Are you now?		
Controlling or losing weight	64.2 (60.4–68.0)	70.4 (66.2–74.6)
Increasing exercise	59.3 (55.8–62.8)	57.9 (51.5–64.3)
Reducing fat intake	58.1 (53.6–62.6)	66.3 (61.4–71.2)

*BF* body fat, *CI* confidence interval, *OLLMM* obesity with low lean muscle mass

### Sensitivity analysis

When using BMI ≥ 27 kg/m^2^ to define obesity instead of percentage BF, combined with the definition of low lean muscle mass, fewer cases of OLLMM (*n* = 671, representing 23,934,214 individuals) were identified. Among the OLLMM cases identified by percentage BF criteria, only 80.3% met the criteria of low lean muscle mass and BMI ≥ 27 kg/m^2^. However, 96.4% of OLLMM cases identified using BMI were also identified based on the percentage BF criteria. Overall, 83.7% of people did not meet either of the criteria for OLLMM (weighted *n* = 151,575,945; *n* = 3307).

The prevalence of OLLMM in the sensitivity analysis was lower with an estimated 13.2% of the USA population (23,934,214 people) during 2017–2018 having OLLMM (Supplemental Figure S3). Although the estimates were consistently lower than the primary analysis, similar trends in the prevalence of OLLMM by sex, age, race/ethnicity, and clinical conditions/procedures were observed in the sensitivity analysis.

## Discussion

Global prevalence estimates of sarcopenic obesity are wide-ranging due to heterogeneity in definitions for sarcopenia and obesity, their measurements, and the populations assessed [6, 15, 25, 26, 31]. By using OLLMM as a standard term and providing clear criteria for both obesity and “low lean muscle mass,” the prevalence of this serious condition was estimated and also stratified across younger adult (aged ≥ 20 years) and elderly (aged ≥ 70 years) age groups, and across BMI classes. Overall, the prevalence of OLLMM was high in the USA, particularly in older age groups. Participants with a higher BMI, prediabetes, T2DM, post-bariatric surgery, or NAFLD with fibrosis had a higher risk of OLLMM, regardless of age.

The prevalence of OLLMM was estimated using 2017–2018 NHANES data for all adults aged ≥ 20 years. Prevalence estimates were determined using either percentage BF or BMI to define obesity. Both definitions found consistent patterns by age, although OLLMM prevalence estimates based on BMI ≥ 27 kg/m^2^ were lower than estimates based on percentage BF, which is consistent with previous studies [28, 29]. This is possibly due to a lack of sensitivity of BMI to reflect an individual's actual percentage BF. Therefore, estimates using BMI rather than percentage BF may underestimate the prevalence of OLLMM, with greater disparity observed in older populations. Compared with previous prevalence estimates using NHANES data from prior survey years in those aged 20–59 years (ranged from 3.3–5.5% [27, 29]) and ≥ 60 years (ranged from 7.0–27.3% [28, 29, 41]), our estimates were generally higher [27–29]. This may be due to an increase in the overall prevalence of disease, variability in the definition, or greater sensitivity of the methodology used in this analysis. The change in prevalence of OLLMM from 1999–2006 to 2017–2018, an increase of 5.5% since 1999–2006 [41], may reflect changes in lifestyle (including diet and physical activity) over time, as well as an overall increase in aging and obese populations since the 1999–2006 NHANES USA study.

Prevalence of OLLMM was consistently higher in participants with T2DM, prediabetes, post-bariatric surgery, and NAFLD with fibrosis. OLLMM prevalence also increased with increasing BMI. These findings are expected and comparable to previous studies, which found higher prevalence in those with a higher BMI, T2DM [16, 42, 43], or NAFLD [30]. Additionally, our findings support prior research highlighting the important role of low muscle mass as a risk factor for metabolic disease in participants with chronic diseases, such as diabetes and chronic kidney disease [16, 44, 45]. Furthermore, the presence of OLLMM may occur as a result of increased inflammation, insulin resistance, and decreased anabolic hormones, all of which are associated with both obesity and low muscle mass [46–48]. In those aged < 60 years, low lean muscle mass was also associated with an increased risk of dysglycemia in both non-obese and obese individuals. In younger as well as in older adults, low lean muscle mass was also much more prevalent in obese than in non-obese individuals [16]. Therefore, with the global rise in obesity, particularly in children and young adults, our data highlight the increasing age-related prevalence of OLLMM, starting in young adulthood. Interventions aimed at increasing muscle mass in younger ages and preventing loss of muscle mass in older ages, such as activin type II receptor antagonists (e.g., bimagrumab), may have the potential to reduce body fat and improve glycemic control in obese individuals, and thus reduce the risk of T2DM [16, 19, 49].

In other clinical settings, including cachexia, low muscle mass has been shown to dramatically increase the risk of mortality and morbidity, even above the pre-existing serious clinical condition [50]. In those aged 70–79 years, sarcopenia or loss of muscle function have been shown to cause a decrease in walking speed, which itself increases mortality/morbidity due to falls and other mobility-related incidences [51]. Thus, in the future it is vital to determine whether low lean muscle mass increases morbidity or mortality in individuals with obesity.

One limitation of the study is the cross-sectional nature of the data, so inferences regarding an increased risk of OLLMM in certain subgroups cannot be made and are outside of the scope of this analysis. Another limitation was the lack of available DXA measures for the age group ≥ 60 years in the 2017–2018 NHANES survey; however, using modeling techniques we were able to estimate the prevalence in this population. The OLLMM definition used in this study relies on body composition; however, several working groups suggest including a measure of strength or function in defining sarcopenia for future studies. We were unable to include this type of measure as it is not included in the most recent version of NHANES. However, the DXA measures of body composition used are robust, whereas measures of strength and function may be less accurate as they are related to the individual’s motivation, and the level of physical activity decreases in older age groups [52]. Furthermore, the FNIH cut points used in this study were previously derived from large clinical datasets such that the cut points discriminated the presence or absence of weakness and slow gait speed [35, 53].

## Conclusions

The prevalence of OLLMM in the USA is higher than previously reported. Individuals with a higher BMI, prediabetes, T2DM, post-bariatric surgery, or NAFLD with fibrosis have a higher risk of OLLMM, regardless of age. A unified definition of OLLMM will help define populations for future clinical trials, especially those aiming to understand the risk of sarcopenia in individuals with obesity, as well as aiding in diagnosis and development of treatment strategies [54]. Clinicians should monitor patients in high-risk clinical groups for OLLMM to facilitate early intervention.

## Supplementary Information

**Additional file 1: Supplemental Table S1.** Criteria of clinical subgroups. **Supplemental Table S2.** Model beta values for participants aged ≥ 60 years. **Supplemental Table S3.** Patient characteristics based on ALM and percentage BF. **Supplemental Table S4.** Functional characteristics of participants by age, with and without OLLMM (based on percentage BF). **Supplemental Figure S1.** Fitted logistic regression model on 70% training sample for OLLMM in (A) males aged ≥ 60 years, and (B) females aged ≥ 60 years. **Supplemental Figure S2.** Performance of selected logistic regression models in 1999–2006 NHANES data, 70% training sample for (A) males aged ≥ 60 years, and (B) females aged ≥ 60 years. **Supplemental Figure S3.** Overall prevalence of OLLMM in the USA identified during 2017–2018 based on BMI ≥ 27 kg/m^2^.

## Data Availability

Publicly available datasets were analyzed in this study. These data can be found at https://www.cdc.gov/nchs/nhanes/index.htm

## Abbreviations

ALM: Appendicular lean mass
AUC: Area under the curve
BF: Body fat
BMI: Body mass index
CI: Confidence interval
DXA: Dual-energy X-ray absorptiometry
FNIH: Foundation of National Institutes of Health
IQR: Interquartile range
NAFLD: Non-alcoholic fatty liver disease
NHANES: National Health and Nutrition Examination Survey
OLLMM: Obesity with low lean muscle mass
ROC: Receiver operating characteristic
T2DM: Type 2 diabetes mellitus
USA: United States of America
