# Supplementary material for: The prevalence of low muscle mass associated with obesity in the USA
Source: Skelet Muscle. 2022 Dec 21;12:26. doi: 10.1186/s13395-022-00309-5 (PMC9769063; doi:10.1186/s13395-022-00309-5)
Supplement: Supplementary file 1 — Additional file 1: Supplemental Table S1. Criteria of clinical subgroups. Supplemental Table S2. Model beta values for participants aged ≥ 60 years. Supplemental Table S3. Patient characteristics based on ALM and percentage BF. Supplemental Table S4. Functional characteristics of participants by age, with and without OLLMM (based on percentage BF). Supplemental Figure S1. Fitted logistic regression model on 70% training sample for OLLMM in (A) males aged ≥ 60 years, and (B) females aged ≥ 60 years. Supplemental Figure S2. Performance of selected logistic regression models in 1999–2006 NHANES data, 70% training sample for (A) males aged ≥ 60 years, and (B) females aged ≥ 60 years. Supplemental Figure S3. Overall prevalence of OLLMM in the USA identified during 2017–2018 based on BMI ≥ 27 kg/m2. [file 13395_2022_309_MOESM1_ESM.docx]

# Supplemental Material

## Methods

## Model performance

A total of 4889 participants aged ≥60 years from the 1999–2006 NHANES database were included in the development of the prediction model, of whom 2455 were females and 2434 were males; there were 1411 from the 1999–2000 wave, 1348 from the 2001–2002 wave, 1546 from the 2003–2004 wave, and 584 from the 2005–2006 wave. Model performance for obesity with low lean muscle mass based on appendicular lean mass (ALM) + body fat was excellent in both males and females aged ≥60 years in training data (model for males: area under the curve (AUC)=0.937, model for females: AUC=0.932) but decreased slightly in testing samples (males: AUC=0.914, females: AUC=0.873), suggesting the fitted model generalizes well to the testing data and over-fitting is unlikely (**Supplemental Figures 1 and 2**). Similarly, model performance for ALM+body mass index in training sample was excellent and remained excellent in the testing sample (males: AUC=0.940 in training data, AUC=0.921 in testing data; females: AUC=0.929 in training data and 0.879 in testing data).

Supplemental Table 1: Criteria of clinical subgroups

| **Group** | **Criteria** |
| --- | --- |
| Obese BMI class | Class 1: BMI of 30–<35  Class 2: BMI of 35–<40  Class 3: BMI of 40 or higher |
| Post-bariatric surgery | Those who answer “yes” to WHQ190 – “Have you ever had weight loss surgery?” |
| T2DM | HbA1c ≥6.5%  OR answer yes to: “Other than during pregnancy, have you ever been told by a doctor or health professional that you have diabetes or sugar diabetes?” (DIQ010=1) (excludes those with who report only insulin therapy and are diagnosed with diabetes at an age <30 years as they are likely to have T1DM) [1] |
| Prediabetes | HbA1c: ≥5.7% and <6.5% OR answer yes to “Have you ever been told by a doctor or other health professional that you have any of the following: prediabetes, impaired fasting glucose, impaired glucose tolerance, or borderline diabetes, or that your blood sugar is higher than normal but not high enough to be called diabetes or sugar diabetes?” (excludes those who report only insulin therapy and are diagnosed with diabetes at an age <30 years as they are likely to have T1DM) [1] |
| NAFLD with fibrosis | LUXCAPM - suspected NAFLD (≥S1) as CAP > 285 dB/m and liver stiffness of ≥8 kPa (≥F2)= fibrosis  Note: for this subgroup we will exclude those with excessive alcohol consumption (>42 g/day for males, >28 g/day for females or consumption of >3 alcoholic drinks per day in males and >2 alcoholic drinks per day in females) [2], or who have aspartate aminotransferase or alanine aminotransferase >500 U/L.  Excessive alcohol consumption was determined using: ALQ130 - Avg # alcohol drinks/day - past 12 months |

ALQ, alcohol use questionnaire; Avg, average; BMI, body mass index; CAP, controlled attenuation parameter; DIQ, diabetes questionnaire; HbA1c, hemoglobin A1c; LUXCAPM, liver ultrasound transient elastography median controlled attenuated parameter; NAFLD, non-alcoholic fatty liver disease; T1DM, type 1 diabetes mellitus; T2DM, type 2 diabetes mellitus.

Supplemental Table 2. Model beta values for participants aged ≥60 years

| **Male aged ≥60 years** | | | | | | | | | |
| --- | --- | --- | --- | --- | --- | --- | --- | --- | --- |
| **varlabel** | **Estimate** | **SE** | **LCLMean** | **UCLMean** | **tValue** | **Probt** | **OR** | **ORLCL** | **ORUCL** |
| Intercept | -10.800301 | 6.864333 | -24.2584 | 2.65779 | -1.57 | 0.1157 |  |  |  |
| Arm Circumference - per 1 cm | -0.252747 | 0.48417 | -1.21519 | 0.70969 | -0.52 | 0.603 | 0.78 | 0.30 | 2.03 |
| Arm Circumference squared | 0.003081 | 0.007426 | -0.01172 | 0.01789 | 0.41 | 0.6794 | 1.00 | 0.99 | 1.02 |
| Body Mass Index - per 1 unit | 1.394137 | 0.409418 | 0.59104 | 2.19723 | 3.41 | 0.0007 | 4.03 | 1.81 | 9.00 |
| Body Mass Index squared | -0.005263 | 0.006962 | -0.01893 | 0.0084 | -0.76 | 0.4498 | 0.99 | 0.98 | 1.01 |
| Waist Circumference-per 10 cm | -0.083971 | 0.928647 | -1.9303 | 1.76236 | -0.09 | 0.9282 | 0.92 | 0.15 | 5.83 |
| Waist Circumference squared | 0.025824 | 0.022488 | -0.01898 | 0.07063 | 1.15 | 0.2545 | 1.03 | 0.98 | 1.07 |
| Weight-per 1 kg | -0.412625 | 0.026922 | -0.4657 | -0.35955 | -15.33 | <.0001 | 0.66 | 0.63 | 0.70 |
| Told to take prescription for cholesterol | 0.262004 | 0.210601 | -0.15191 | 0.67592 | 1.24 | 0.2141 | 1.30 | 0.86 | 1.97 |
| Told had high blood pressure - 2+ times | -0.311045 | 0.333468 | -0.96483 | 0.34274 | -0.93 | 0.351 | 0.73 | 0.38 | 1.41 |
| Taking prescription for hypertension | 0.497323 | 0.333597 | -0.15666 | 1.15131 | 1.49 | 0.1361 | 1.64 | 0.85 | 3.16 |
| Doctor told you have diabetes | -0.493585 | 0.416977 | -1.31122 | 0.32405 | -1.18 | 0.2366 | 0.61 | 0.27 | 1.38 |
| Take diabetic pills to lower blood sugar | 0.396667 | 0.448391 | -0.48317 | 1.2765 | 0.88 | 0.3765 | 1.49 | 0.62 | 3.58 |
| HDL-Cholesterol - per 0.1 mmol/L | -0.055503 | 0.110988 | -0.27308 | 0.16207 | -0.5 | 0.617 | 0.95 | 0.76 | 1.18 |
| HDL-Cholesterol squared | 0.003099 | 0.003616 | -0.00399 | 0.01019 | 0.86 | 0.3915 | 1.00 | 1.00 | 1.01 |
| Glycohemoglobin - per 1 percent | 0.144071 | 0.089718 | -0.03263 | 0.32077 | 1.61 | 0.1096 | 1.15 | 0.97 | 1.38 |
| Ever told you had angina/angina pectoris | 0.458369 | 0.321052 | -0.17157 | 1.08831 | 1.43 | 0.1537 | 1.58 | 0.84 | 2.97 |
| Ever been told you have asthma | -0.289476 | 0.325672 | -0.92842 | 0.34947 | -0.89 | 0.3743 | 0.75 | 0.40 | 1.42 |
| Ever told you had bronchitis | 0.818444 | 0.387217 | 0.05947 | 1.57742 | 2.11 | 0.0346 | 2.27 | 1.06 | 4.84 |
| Ever told had congestive heart failure | 0.526722 | 0.324746 | -0.10987 | 1.16331 | 1.62 | 0.1049 | 1.69 | 0.90 | 3.20 |
| Ever told you had heart attack | -0.231016 | 0.278063 | -0.77694 | 0.31491 | -0.83 | 0.4064 | 0.79 | 0.46 | 1.37 |
| Ever told you had any liver condition | 1.288659 | 0.488243 | 0.33167 | 2.24565 | 2.64 | 0.0083 | 3.63 | 1.39 | 9.45 |
| Ever told you had a stroke | 0.387658 | 0.338364 | -0.27932 | 1.05463 | 1.15 | 0.2532 | 1.47 | 0.76 | 2.87 |
| Walking between rooms on same floor: some/much difficulty/unable to do | -0.989035 | 0.447062 | -1.87272 | -0.10535 | -2.21 | 0.0285 | 0.37 | 0.15 | 0.90 |
| Standing up from armless chair: some/much difficulty/unable to do | 0.675499 | 0.345981 | -0.00473 | 1.35573 | 1.95 | 0.0516 | 1.97 | 1.00 | 3.88 |
| Dressing yourself: some/much difficulty/unable to do | -0.394057 | 0.351108 | -1.08277 | 0.29465 | -1.12 | 0.2619 | 0.67 | 0.34 | 1.34 |
| Need special equipment to walk | 0.534163 | 0.424513 | -0.2982 | 1.36652 | 1.26 | 0.2084 | 1.71 | 0.74 | 3.92 |
| House chore: some/much difficulty/unable to do | -0.568015 | 0.358925 | -1.27205 | 0.13602 | -1.58 | 0.1137 | 0.57 | 0.28 | 1.15 |
| Limitations keeping you from working | 0.657265 | 0.367932 | -0.0641 | 1.37863 | 1.79 | 0.0741 | 1.93 | 0.94 | 3.97 |
| Managing money: some/much difficulty/unable to do | 0.540185 | 0.351489 | -0.15085 | 1.23122 | 1.54 | 0.1251 | 1.72 | 0.86 | 3.43 |
| Reaching up over head: some/much difficulty/unable to do | -0.7286 | 0.333512 | -1.38478 | -0.07242 | -2.18 | 0.0297 | 0.48 | 0.25 | 0.93 |
| Stooping/crouching/kneeling: much difficulty/unable to do | 0.558899 | 0.36115 | -0.14981 | 1.26761 | 1.55 | 0.122 | 1.75 | 0.86 | 3.55 |
| Walking up ten steps: some/much difficulty/unable to do | 0.233719 | 0.455598 | -0.65934 | 1.12677 | 0.51 | 0.608 | 1.26 | 0.52 | 3.09 |
| Walking up ten steps: much difficulty/unable to do | 0.929862 | 0.649261 | -0.35136 | 2.21108 | 1.43 | 0.1538 | 2.53 | 0.70 | 9.13 |
| Limited in amount of work you can do | -0.613349 | 0.346739 | -1.29314 | 0.06644 | -1.77 | 0.077 | 0.54 | 0.27 | 1.07 |
| Age - per 1 year increase | 0.050901 | 0.013543 | 0.02434 | 0.07747 | 3.76 | 0.0002 | 1.05 | 1.02 | 1.08 |
| Black (ref: White) | -2.20857 | 0.36097 | -2.91802 | -1.49912 | -6.12 | <.0001 | 0.11 | 0.05 | 0.22 |
| Mexican (ref: White) | 0.068387 | 0.215884 | -0.35492 | 0.49169 | 0.32 | 0.7514 | 1.07 | 0.70 | 1.64 |
| Other race (ref: White) | -0.095746 | 0.477914 | -1.03248 | 0.84098 | -0.2 | 0.8412 | 0.91 | 0.36 | 2.32 |
| Other Hispanic (ref: White) | -0.773297 | 0.503699 | -1.76059 | 0.214 | -1.54 | 0.1247 | 0.46 | 0.17 | 1.24 |
| **Female aged ≥60 years** | | | | | | | | | |
| **varlabel** | **Estimate** | **SE** | **LCLMean** | **UCLMean** | **tValue** | **Probt** | **OR** | **ORLCL** | **ORUCL** |
| Intercept | -18.085273 | 5.639608 | -29.175 | -6.99555 | -3.21 | 0.0015 |  |  |  |
| Arm Circumference - per 1 cm | 0.188227 | 0.26883 | -0.33888 | 0.71533 | 0.7 | 0.4839 | 1.21 | 0.71 | 2.04 |
| Arm Circumference squared | -0.0031 | 0.003898 | -0.01074 | 0.00454 | -0.8 | 0.4265 | 1.00 | 0.99 | 1.00 |
| Body Mass Index - per 1 unit | 1.606932 | 0.293331 | 1.03006 | 2.18381 | 5.48 | <.0001 | 4.99 | 2.80 | 8.88 |
| Body Mass Index squared | -0.010145 | 0.004361 | -0.01871 | -0.00158 | -2.33 | 0.0203 | 0.99 | 0.98 | 1.00 |
| Waist Circumference-per 10 cm | 0.386013 | 0.522003 | -0.63729 | 1.40932 | 0.74 | 0.4596 | 1.47 | 0.53 | 4.09 |
| Waist Circumference squared | -0.004118 | 0.012865 | -0.02934 | 0.0211 | -0.32 | 0.7489 | 1.00 | 0.97 | 1.02 |
| Weight-per 1 kg | -0.502987 | 0.101464 | -0.70248 | -0.30349 | -4.96 | <.0001 | 0.60 | 0.50 | 0.74 |
| Weight squared | 0.00098 | 0.000641 | -0.00028 | 0.00224 | 1.53 | 0.1272 | 1.00 | 1.00 | 1.00 |
| Told to take prescription for cholesterol | -0.18362 | 0.185316 | -0.54717 | 0.17993 | -0.99 | 0.3219 | 0.83 | 0.58 | 1.20 |
| Told had high blood pressure - 2+ times | -0.297194 | 0.314156 | -0.913 | 0.31862 | -0.95 | 0.3442 | 0.74 | 0.40 | 1.38 |
| Taking prescription for hypertension | 0.423411 | 0.313801 | -0.19166 | 1.03848 | 1.35 | 0.1773 | 1.53 | 0.83 | 2.82 |
| Taking insulin now | 0.561866 | 0.403507 | -0.22944 | 1.35317 | 1.39 | 0.1639 | 1.75 | 0.79 | 3.87 |
| HDL-Cholesterol - per 0.1 mmol/L | 0.375233 | 0.146811 | 0.07269 | 0.67777 | 2.56 | 0.0171 | 1.46 | 1.08 | 1.97 |
| HDL-Cholesterol squared | -0.009278 | 0.004401 | -0.01841 | -0.00015 | -2.11 | 0.0467 | 0.99 | 0.98 | 1.00 |
| Glycohemoglobin - per 1 percent | -0.497601 | 0.323809 | -1.13281 | 0.1376 | -1.54 | 0.1246 | 0.61 | 0.32 | 1.15 |
| Glycohemoglobin squared | 0.026906 | 0.018499 | -0.00938 | 0.06319 | 1.45 | 0.146 | 1.03 | 0.99 | 1.07 |
| Total cholesterol - per 1 mmol/L | 0.005107 | 0.006996 | -0.00868 | 0.01889 | 0.73 | 0.4661 | 1.01 | 0.99 | 1.02 |
| Ever told you had angina/angina pectoris | 0.357575 | 0.29701 | -0.22459 | 0.93974 | 1.2 | 0.2286 | 1.43 | 0.80 | 2.56 |
| Ever told you had heart attack | 1.165751 | 0.317311 | 0.54274 | 1.78877 | 3.67 | 0.0003 | 3.21 | 1.72 | 5.98 |
| Dressing yourself: some/much difficulty/unable to do | -0.431002 | 0.439393 | -1.31896 | 0.45696 | -0.98 | 0.3325 | 0.65 | 0.27 | 1.58 |
| Dressing yourself: much difficulty/unable to do | 0.478496 | 0.607347 | -0.71717 | 1.67417 | 0.79 | 0.4315 | 1.61 | 0.49 | 5.33 |
| House chore: much difficulty/unable to do | -0.62093 | 0.397974 | -1.40198 | 0.16012 | -1.56 | 0.1191 | 0.54 | 0.25 | 1.17 |
| Lifting or carrying: some/much difficulty/unable to do | 0.335274 | 0.344745 | -0.34284 | 1.01339 | 0.97 | 0.3315 | 1.40 | 0.71 | 2.75 |
| Lifting or carrying: much difficulty/unable to do | -0.259576 | 0.389781 | -1.02446 | 0.50531 | -0.67 | 0.5056 | 0.77 | 0.36 | 1.66 |
| Managing money: some/much difficulty/unable to do | -0.639684 | 0.463762 | -1.54924 | 0.26988 | -1.38 | 0.168 | 0.53 | 0.21 | 1.31 |
| Managing money: much difficulty/unable to do | -0.47702 | 0.666907 | -1.78544 | 0.8314 | -0.72 | 0.4746 | 0.62 | 0.17 | 2.30 |
| Reaching up over head: some/much difficulty/unable to do | -0.721925 | 0.373376 | -1.45668 | 0.01283 | -1.93 | 0.0541 | 0.49 | 0.23 | 1.01 |
| Reaching up over head: much difficulty/unable to do | 0.67379 | 0.513 | -0.34098 | 1.68856 | 1.31 | 0.1913 | 1.96 | 0.71 | 5.41 |
| Standing for long periods: much difficulty/unable to do | 0.656452 | 0.324357 | 0.01896 | 1.29394 | 2.02 | 0.0436 | 1.93 | 1.02 | 3.65 |
| Walking up ten steps: some/much difficulty/unable to do | 0.257497 | 0.322848 | -0.37528 | 0.89028 | 0.8 | 0.4251 | 1.29 | 0.69 | 2.44 |
| Walking for a quarter mile: much difficulty/unable to do | 0.157261 | 0.36465 | -0.5575 | 0.87203 | 0.43 | 0.6663 | 1.17 | 0.57 | 2.39 |
| Limited in amount of work you can do | 0.276473 | 0.256772 | -0.22684 | 0.77979 | 1.08 | 0.2816 | 1.32 | 0.80 | 2.18 |
| Age - per 1y increase | -0.016004 | 0.012282 | -0.04008 | 0.00807 | -1.3 | 0.1926 | 0.98 | 0.96 | 1.01 |
| Black (ref: White) | -2.311167 | 0.354333 | -3.0057 | -1.61664 | -6.52 | <.0001 | 0.10 | 0.05 | 0.20 |
| Mexican (ref: White) | 0.587297 | 0.209124 | 0.17734 | 0.99726 | 2.81 | 0.005 | 1.80 | 1.19 | 2.71 |
| Other race (ref: White) | -0.857426 | 0.615203 | -2.06565 | 0.3508 | -1.39 | 0.1639 | 0.42 | 0.13 | 1.42 |
| Other Hispanic (ref: White) | -0.38699 | 0.388114 | -1.14768 | 0.3737 | -1 | 0.3187 | 0.68 | 0.32 | 1.45 |

HDL, high-density lipoprotein; LCL, lower confidence limits; Probt, probability; OR, odds ratio; SE, standard error; UCL, upper confidence limits; y, year.

Supplemental Table 3. Patient characteristics based on ALM and percentage BF

| **Variable** | **Without OLLMM** | **OLLMM** |
| --- | --- | --- |
| Participants, weighted (unweighted) | 152,448,177 (3,347) | 28,728,420 (827) |
| Demographic and clinical characteristics |  |  |
| Males, % (95% CI) | 48.3 (46.4–50.3) | 46.2 (40.7–51.7) |
| Age at screening, years, median (IQR) | 47.9 (32.6–62.5) | 65.2 (54.9–74.8) |
| Ethnicity/Hispanic origin, % (95% CI) |  |  |
| Non-Hispanic White | 63.8 (57.8–69.8) | 64.6 (56.5–72.7) |
| Non-Hispanic Black | 11.3 (7.7–14.9) | 2.3 (1.3–3.4) |
| Mexican-American | 7.4 (4.1–10.7) | 14.5 (8.9–20.0) |
| Other Hispanic | 6.6 (4.8–8.4) | 9.4 (6.4–12.3) |
| Other race/ethnicity, including multi-racial | 10.9 (8.0–13.7) | 9.2 (4.9–13.6) |
| Subgroups of interest, % (95% CI) |  |  |
| T2DM | 11.4 (9.6–13.1) | 31.8 (27.6–36.1) |
| Prediabetes | 21.7 (19.3–24.0) | 28.1 (23.3–33.0) |
| NAFLD with fibrosis | 3.0 (2.2–3.8) | 6.9 (4.3–9.6) |
| History of bariatric or weight loss surgery | 1.3 (0.4–2.2) | 1.9 (0.6–3.3) |
| Obesity, % (95% CI) |  |  |
| Class 1: BMI of 30–<35 | 21.1 (17.9–24.3) | 29.8 (25.1–34.5) |
| Class 2: BMI of 35–<40 | 8.7 (7.2–10.1) | 21.7 (16.4–27.0) |
| Class 3: BMI of ≥40 | 4.9 (3.4–6.3) | 13.8 (10.1–17.4) |
| Weight, kg, median (IQR) | 77.4 (66.2–93.0) | 83.3 (70.2–96.9) |
| BMI, kg/m^2^, median (IQR) | 27.4 (23.7–31.7) | 32.6 (28.6–36.3) |
| Waist circumference, cm, median (IQR) | 96.2 (85.7–107.4) | 108.9 (100.0–118.9) |

ALM, appendicular lean mass; BF, body fat; BMI, body mass index; CI, confidence interval; IQR, interquartile range; NAFLD, non-alcoholic fatty liver disease; OLLMM, obesity with low lean muscle mass; T2DM, type 2 diabetes mellitus.

Supplemental Table 4. Functional characteristics of participants by age, with and without OLLMM (based on percentage BF)

| Participants, weighted N (n) | **Age 20–59 years** | | **Age ≥60 years** | |
| --- | --- | --- | --- | --- |
|  | **Without OLLMM** | **With OLLMM** | **Without OLLMM** | **With OLLMM** |
|  | 102,580,107 (1926) | 9,000,742 (230) | 49,868,070 (1421) | 19,727,678 (597) |
| Physical characteristics (some/much difficult/unable to do), % (95% CI) | | | | |
| Managing money | 4.9 (3.2–6.5) | 5.8 (1.9–9.7) | 12.5 (10.6–14.4) | 9.6 (5.6–13.7) |
| Walking for a quarter mile | 4.2 (3.5–5.0) | 8.2 (1.8–14.5) | 10.4 (6.8–13.9) | 17.0 (13.5–20.5) |
| Walking up 10 steps | 2.7 (2.0–3.3) | 5.1 (1.7–8.5) | 6.1 (3.9–8.3) | 10.9 (6.4–15.5) |
| Stooping, crouching, or kneeling | 8.5 (6.8–10.2) | 21.2 (13.5–28.8) | 34.2 (27.7–40.7) | 46.3 (38.6–54.1) |
| Lifting or carrying | 6.6 (5.4–7.9) | 11.4 (6.2–16.7) | 21.5 (17.5–25.5) | 28.2 (23.4–33.0) |
| House chores | 6.7 (5.0–8.5) | 16.4 (11.5–21.3) | 25.1 (20.2–30.0) | 31.5 (25.0–38.1) |
| Preparing meals | 2.4 (1.6–3.1) | 5.9 (1.9–9.8) | 13.4 (10.7–16.1) | 14.1 (10.7–17.6) |
| Walking between rooms on the same floor | 2.3 (1.1–3.4) | 7.4 (2.8–12.0) | 12.2 (8.3–16.1) | 11.5 (8.1–15.0) |
| Standing up from an armless chair | 4.4 (3.3–5.5) | 11.3 (5.6–16.9) | 22.5 (17.4–27.5) | 26.0 (21.7–30.3) |
| Getting in and out of bed | 4.6 (3.0–6.2) | 9.9 (5.1–14.7) | 18.1 (13.3–22.9) | 18.3 (14.1–22.5) |
| Using fork or knife, or drinking from cup | 1.0 (0.4–1.6) | 3.3 (0.0–6.9) | 5.0 (3.2–6.8) | 4.9 (2.2–7.5) |
| Dressing yourself | 2.9 (1.8–4.1) | 7.3 (3.0–11.7) | 15.2 (11.8–18.5) | 14.7 (9.8–19.5) |
| Standing for long periods | 8.3 (6.8–9.8) | 19.7 (14.5–24.9) | 33.5 (27.9–39.1) | 48.2 (40.1–56.4) |
| Sitting for long periods | 7.1 (5.9–8.4) | 14.4 (8.4–20.3) | 19.3 (15.2–23.5) | 22.4 (18.1–26.7) |
| Reaching up over your head | 5.5 (3.8–7.1) | 8.2 (3.7–12.6) | 16.6 (12.8–20.4) | 22.1 (15.5–28.6) |
| Grasping/holding small objects | 3.2 (2.1–4.4) | 7.0 (3.1–11.0) | 14.5 (9.7–19.4) | 18.5 (13.6–23.5) |

BF, body fat; CI, confidence interval; OLLMM, obesity with low lean muscle mass.

Supplemental Figure 1: Fitted logistic regression model on 70% training sample for OLLMM in (A) males aged ≥60 years, and (B) females aged ≥60 years

**A**
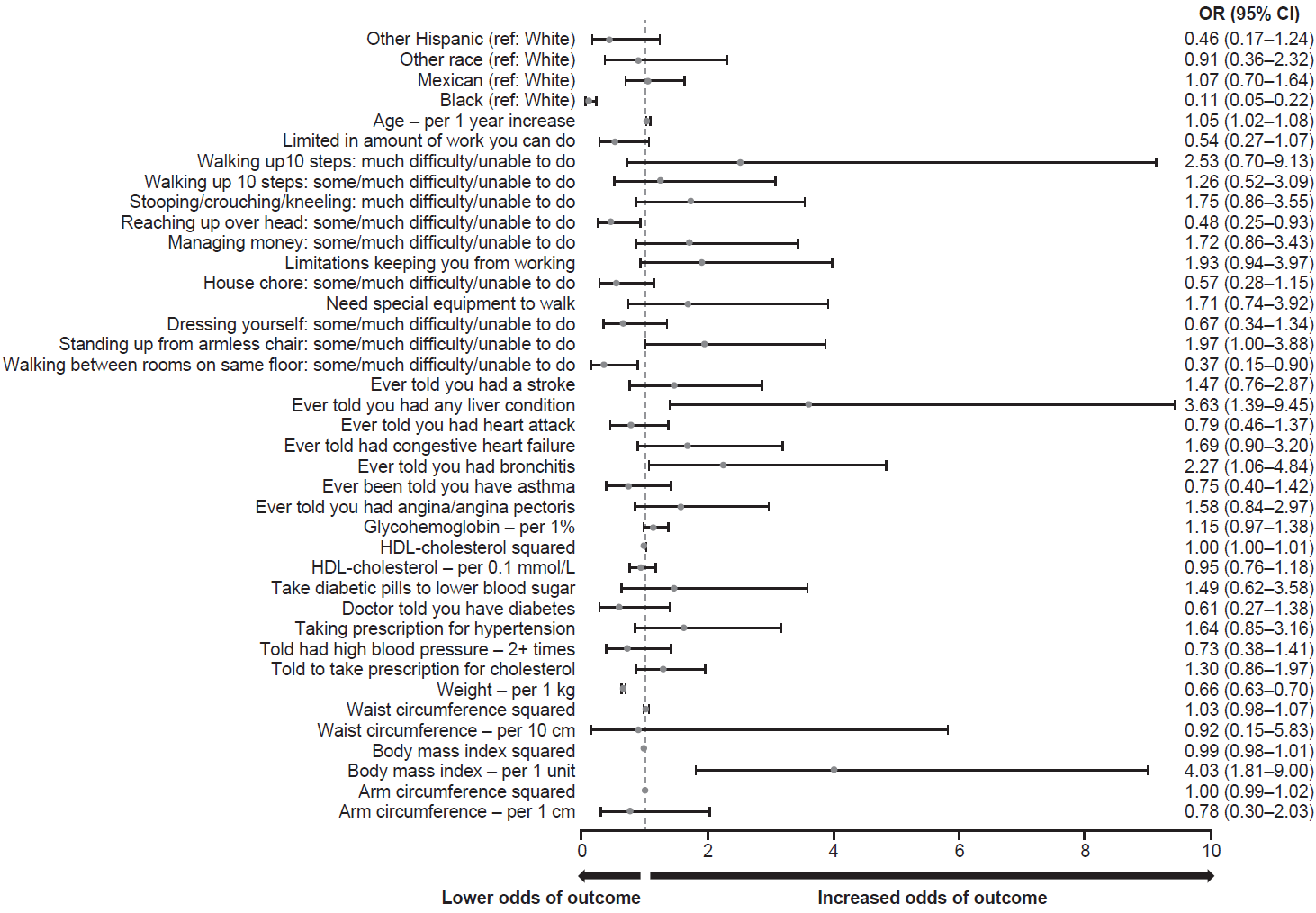


**B**
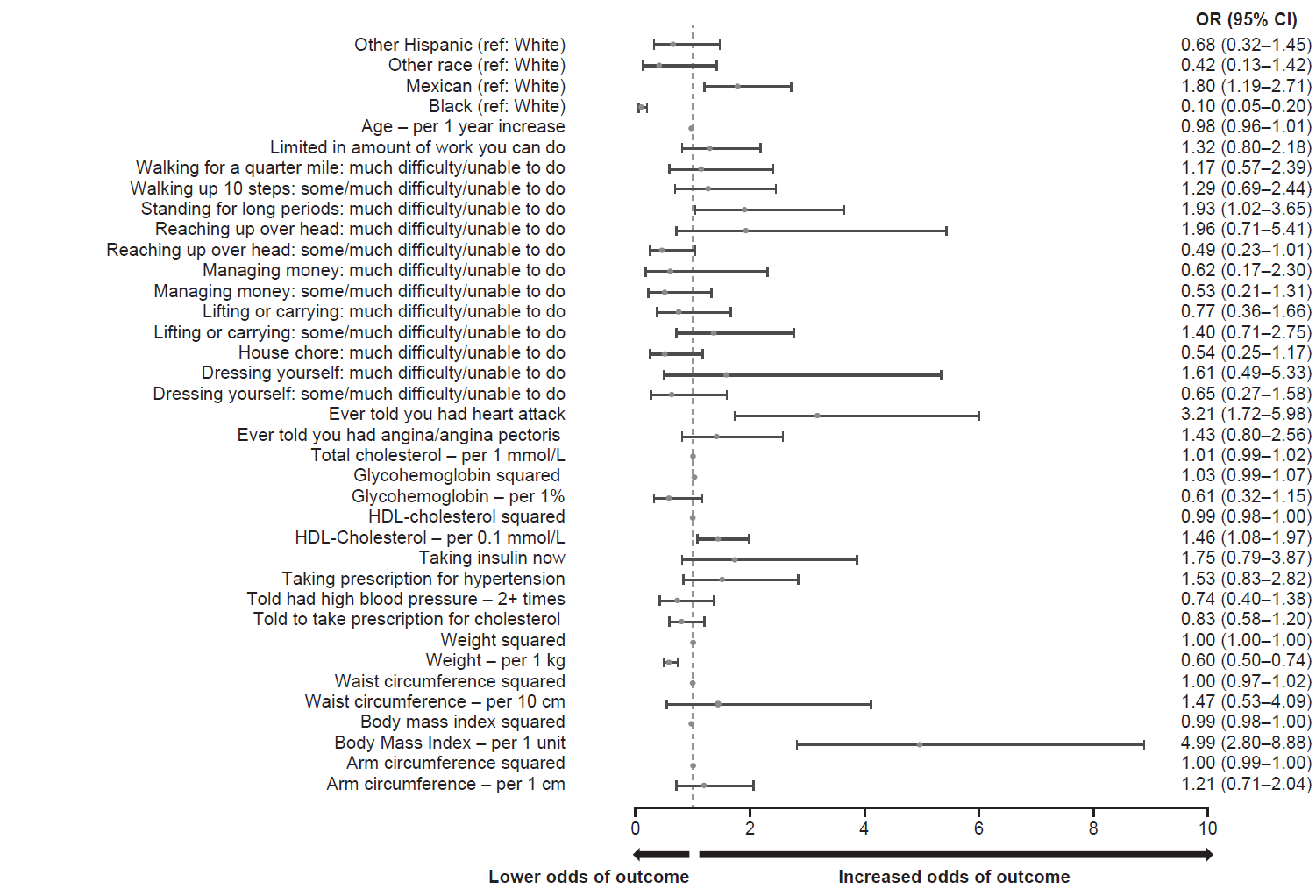


CI, confidence interval; HDL, high-density lipoprotein; OLLMM, obesity with low lean muscle mass.

Supplemental Figure 2. Performance of selected logistic regression models in 1998–2006 NHANES data, 70% training sample for (A) males aged ≥60 years, and (B) females aged ≥60 years

ROC, receiver operating characteristic; NHANES, National Health and Nutrition Examination Survey

Supplemental Figure 3. Overall prevalence of OLLMM in the US identified during 2017–2018 based on BMI ≥27 kg/m^2^


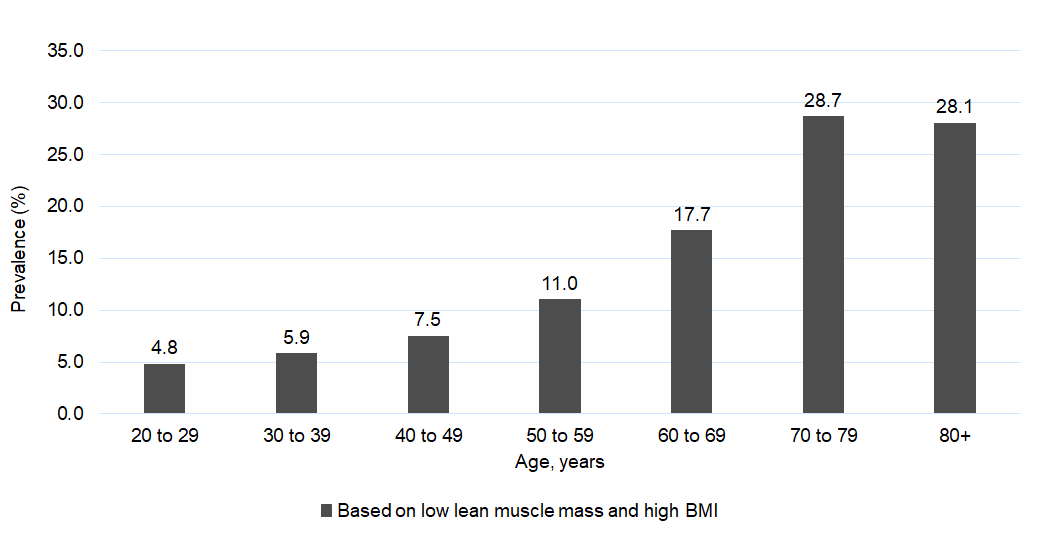


BMI, body mass index; OLLMM, obesity with low lean muscle mass.

1. Koopman RJ, Mainous AG, 3rd, Diaz VA, Geesey ME (2005) Changes in age at diagnosis of type 2 diabetes mellitus in the United States, 1988 to 2000. Ann Fam Med 3(1):60-63. doi:10.1370/afm.214

2. Singal AK, Bataller R, Ahn J, Kamath PS, Shah VH (2018) ACG Clinical Guideline: Alcoholic Liver Disease. Am J Gastroenterol 113(2):175-194. doi:10.1038/ajg.2017.469
